# The lncRNA H19 positively affects the tumorigenic properties of glioblastoma cells and contributes to NKD1 repression through the recruitment of EZH2 on its promoter

**DOI:** 10.18632/oncotarget.24496

**Published:** 2018-02-14

**Authors:** Barbara Fazi, Sabrina Garbo, Nicola Toschi, Annunziato Mangiola, Malinska Lombari, Daria Sicari, Cecilia Battistelli, Silvia Galardi, Alessandro Michienzi, Gianluca Trevisi, Rona Harari-Steinfeld, Carla Cicchini, Silvia Anna Ciafrè

**Affiliations:** ^1^ Department of Biomedicine and Prevention, University of Rome “Tor Vergata”, Rome, Italy; ^2^ Department of Radiology, Athinoula A. Martinos Center for Biomedical Imaging, Boston, MA, USA; ^3^ Harvard Medical School, Boston, MA, USA; ^4^ Department Head and Neck, Institute of Neurosurgery, Catholic University of Sacred Heart, Rome, Italy; ^5^ Department of Cellular Biotechnologies and Haematology, Sezione di Genetica Molecolare, Istituto Pasteur Italia-Fondazione Cenci Bolognetti, Sapienza University of Rome, Rome, Italy; ^6^ Goldyne Savad Institute of Gene Therapy, Hadassah University Hospital, Hebrew University, Jerusalem; ^7^ Present address: Laboratorio Nazionale CIB (LNCIB), AREA Science Park, Trieste, Italy

**Keywords:** glioblastoma, lncRNA, H19, NKD1, EZH2

## Abstract

The still largely obscure molecular events in the glioblastoma oncogenesis, a primary brain tumor characterized by an inevitably dismal prognosis, impel for investigation. The importance of Long noncoding RNAs as regulators of gene expression has recently become evident. Among them, H19 has a recognized oncogenic role in several types of human tumors and was shown to correlate to some oncogenic aspects of glioblastoma cells. Here we, hypothesyze that in glioblastoma H19 exerts its function through the interaction with the catalytic subunit of the PRC2 complex, EZH2. By employing a factor analysis on a SAGE dataset of 12 glioblastoma samples, we show that H19 expression in glioblastoma tissues correlates with that of several genes involved in glioblastoma growth and progression. H19 knock-down reduces viability, migration and invasiveness of two distinct human glioblastoma cell lines. Most importantly, we provide a mechanistic perspective about the role of H19 in glioblastoma cells, by showing that its expression is inversely linked to that of NKD1, a negative regulator of Wnt pathway, suggesting that H19 might regulate NKD1 transcription via EZH2-induced H3K27 trimethylation of its promoter. Indeed, we showed that H19 binds EZH2 in glioblastoma cells, and that EZH2 binding to NKD1 and other promoters is impaired by H19 silencing.

In this work we describe H19 as part of an epigenetic modulation program executed by EZH2, that results in the repression of Nkd1. We believe that our results can provide a new piece to the complex puzzle of H19 function in glioblastoma.

## INTRODUCTION

Long noncoding (lncRNAs) RNAs are noncoding RNA molecules longer than 200 nucleotides that exert several regulatory functions, at both transcriptional and post-transcriptional level [1]. The number of annotated lncRNAs is constantly increasing in almost all species, thanks to the development of measurement techniques (essentially based on Next Generation Sequencing, NGS) that allow for the detection of even very scarcely expressed molecules (as is often the case with lncRNAs). The scientific interest in these molecules peaked at the beginning of the century with the discovery of their role as important regulators of a wide range of physiological and pathological states. Still, a few lncRNAs had been discovered previously, even though only a small share of their function had been elucidated. Among these “older” lncRNAs, H19 is a 2.3 kb “oncofetal” RNA, maternally expressed and paternally imprinted, whose gene is located at 11p15.5, close to the telomeric region of chromosome 11, and is reciprocally imprinted and regulated with its neighboring gene IGF2 [2]. Its expression is high during the embryonic period, but becomes barely detectable at birth and all along adult life, with the significant exception of a notably increased expression in tumors. This observation, along with the results of a growing number of studies, indicates that H19 works as an oncogene in several types of human cancers [3]. In the context of tumor cells, it was shown to work as a chromatin modifier through the recruitment of EZH2 to the promoter of target genes [4], as a competing endogenous RNA, sponging let-7 [5, 6], and also as the precursor for miR-675, in turn involved in tumorigenesis [7–12]. In both its transcriptional and post-transcriptional roles, its expression in cancer cells was shown to be related to tumor dissemination and EMT, and in some cases to drug resistance[13–15].

Glioblastoma multiforme (GBM) is the most frequent and deadliest among malignant brain tumors, with an incidence of more than 3–4 cases per 100,000 per year [16], and is inevitably characterized by a dismal, always lethal prognosis. This remains true in spite of recent advances in surgical techniques and radiotherapy, flanked by adjuvant chemotherapy, which result in a median overall survival time of 12–18 months from diagnosis [17, 18]. The first observations of H19 expression in glioblastoma cells are relatively recent (last five years) and all indicate an oncogenic role of H19 mediated by enhancing migration/invasion, proliferation as well as stemness. The molecular mechanisms described so far for an oncogenic role of H19 in GBM range from H19 processing to produce miR-675 [8, 19] to a function as sponge for miR-29a, in turn boosting tumor angiogenesis [20]. Our recent serial analysis of gene expression (SAGE) profiling of glioblastoma tissues [21] showed that H19 is overexpressed in tumor samples from short-term survivors compared to healthy white matter, but notably also in the same samples when compared to tumor tissues from long-term survivors. In addition, H19 expression clearly differentiates tumor samples from short-term survivors from samples excised from the peritumoral areas of the same patients.

Given the cumulative evidence pointing to H19 as an oncogene in glioblastoma, in this work we aimed to unravel the molecular basis of this role, with a specific focus on its possible involvement in the epigenetic modulation of gene expression mediated by EZH2 binding and trimethylation of H3K27 at regulatory regions.

## RESULTS

### The expression of the lncRNA H19 characterizes glioblastoma tissues and cell lines and correlates with that of several genes involved in glioblastoma growth and progression

**Figure 1:**
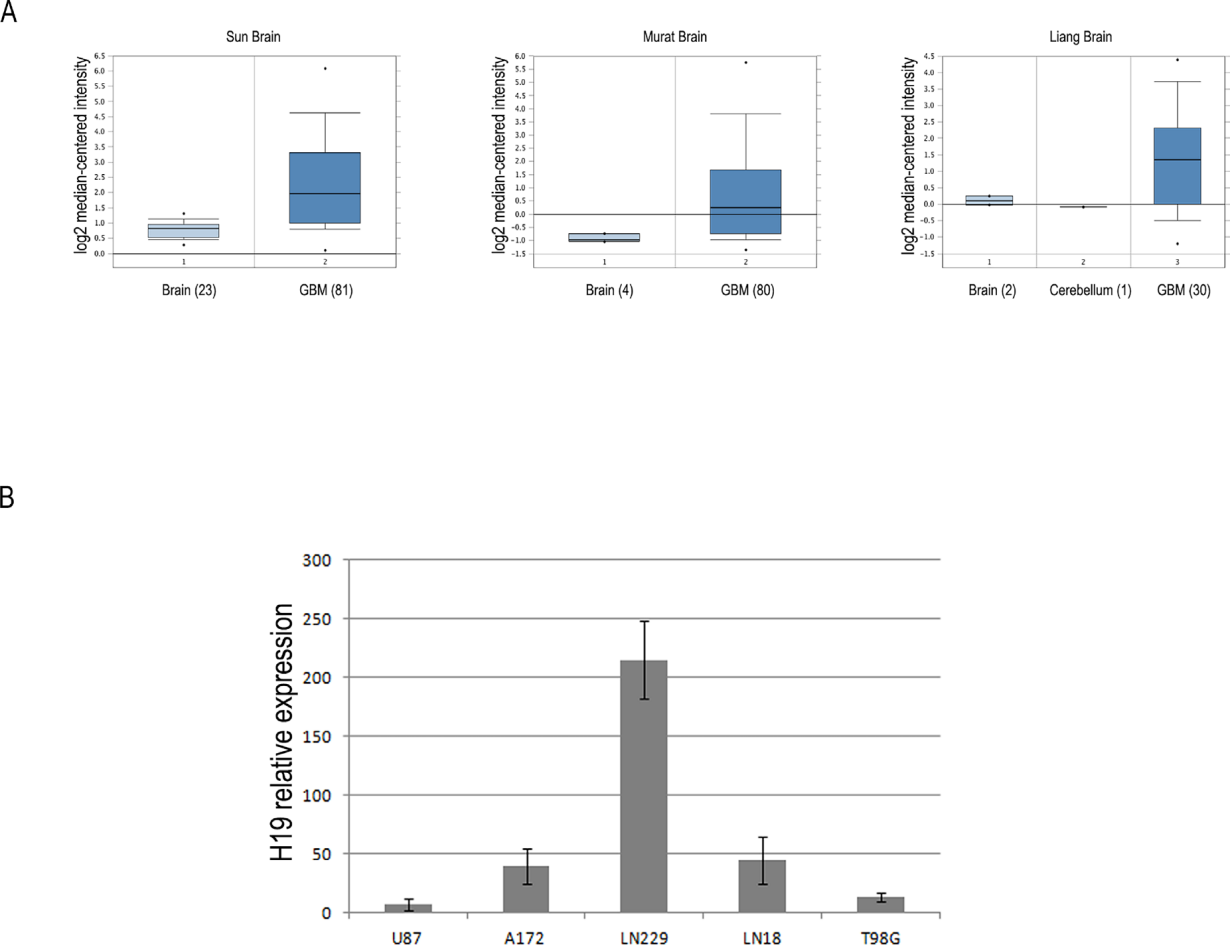
The lncRNA H19 is overexpressed in glioblastoma tissues and cell lines compared to heathy white matter (**A**) Box-plots of relative H19 expression in glioblastoma and normal brain samples in three distinct datasets from Oncomine database (https://www.oncomine.org/). For each group of samples, the number in brackets represents the number of subjects analyzed. The horizontal lines represent the median values and the bottom and the top of the boxes correspond to the 25th and 75th percentiles, respectively. A non-parametric *t* test was performed to calculate *p* values as indicated in the text. (**B**) RT-qPCR analysis of relative H19 expression in five human glioblastoma cell lines. All data are expressed as compared to H19 expression levels measured in a healthy white matter sample, set as = 1.

In order to corroborate our previous findings about high H19 expression in glioblastoma [21], we searched the cancer gene expression database Oncomine (www.oncomine.org) for H19 expression in glioblastomas, and found that it is significantly overexpressed in GBM samples as compared to healthy brain control tissues. Figure 1A depicts H19 expression in three GBM datasets present in Oncomine. In all of them, H19 is in the top 5% of overexpressed genes in GBM *vs* healthy controls and its fold change ranges from 2.4 (Liang dataset, *p* = 8.80E-5) through 2.7 (Sun dataset, *p* = 8.35E-13) to 3.2 (Murat dataset, *p* = 2.13E-11). We then employed a factor analysis approach to study H19 expression in our GBM sample cohort (12 glioblastoma tissues, described in ref.21); in this data reduction approach, a large set of correlated variables is mapped to a smaller set of uncorrelated linear combinations (factors) of the original variables along with the relative contributions (loadings) of each variable to each factor. This yields a small set of uncorrelated variables from a large set of variables (most of which are correlated to each other). Thus, when considering only genes with loadings >0.5, (loadings are expressed with values from 0 to 1, the higher this number, the more that gene contributes to that factor) we found that H19 expression is clearly associated with a single factor (loading = –0.85 on the second factor extracted, which explained 17% of total variance), which also collects other genes strongly involved in GBM development. These include LIF, induced by TGFβ in GBM initiating cells and supporting their self-renewal and migration ability [22], or other genes working in glucose metabolism, as hexokinase 3 (HK3), or in detoxification of ROS, as glutathione peroxidase 3 (GPX3) [23]. These are all processes perturbed in GBM and in many cases necessary for its growth and spreading. All genes with loadings greater than 0.5 on the same factor as H19 are shown in Table 1. Complete factor analysis results (in terms of loadings) are shown in Supplementary Table 1.

**Table 1:** Genes with loadings greater than 0.5 (in absolute value) on the same factor on which H19 was found to have a loading of –0.85

Gene	Loadings
CDCP1	−0.952807
CA3	−0.870870
H19	−0.857627
SLN	−0.843568
TGFBI	−0.800498
LIF	−0.769733
GPX3	−0.717141
SCNN1B	−0.703187
OCIAD2	−0.693885
HK3	−0.620037
SHCBP1	−0.563494

Successively, with the aim of establishing an *in vitro* system where studying the role of H19 in GBM, we checked H19 expression levels in 5 human glioblastoma cell lines by comparing them with a healthy white matter sample. As shown in Figure 1B, the LN229 and A172 cell lines are those with the highest expression of H19. For this reason, we chose these two cell lines to perform our experiments of H19 knock down.

### The lncRNA H19 affects viability, migration and invasiveness of glioblastoma cells

**Figure 2:**
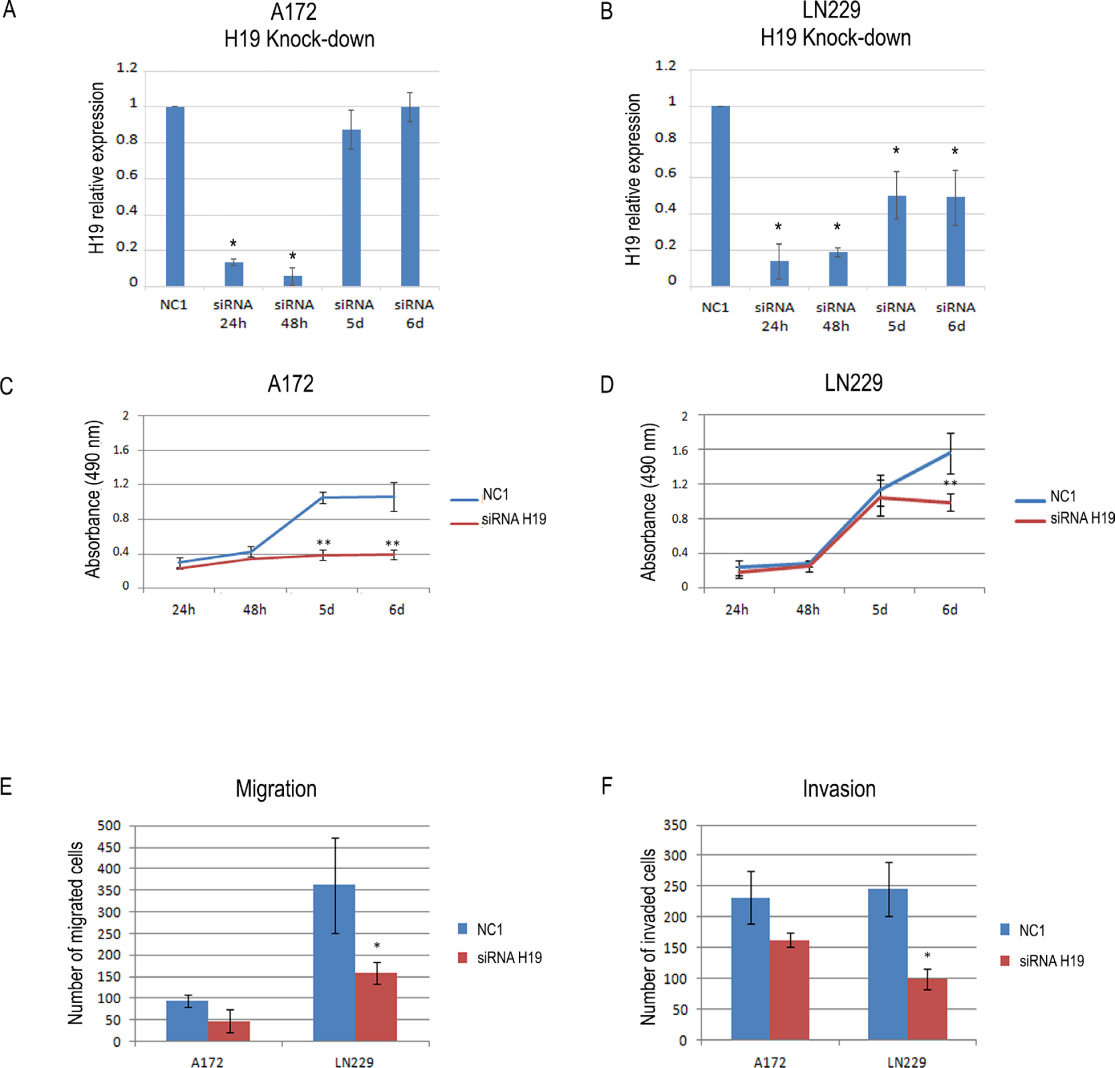
The knock-down of H19 reduces the viability and the migration and invasion ability of human glioblastoma cells (**A**–**B**) Time course of H19 knock-down in A172 (a) and LN229 (b) human glioblastoma cells. The graph shows the results of RT-qPCR analysis of H19 relative expression in cells transfected with either a non-targeting, negative control siRNA (NC1) or a siRNA specifically targeting H19. At all the time points, H19 expression in siRNA H19-transfected cells is shown as compared to that of NC1-transfected cells, set as = 1. (**C**–**D**) Viability (MTS) assays performed on A172 (C) and LN229 (D) cells transfected with either a negative control (NC1) or a H19 targeting siRNA, as in (A–B). Results in a-d are shown as the mean ± S.D. and represent the average of three experiments performed independently. Data were analyzed by a two-tailed unpaired Student’s *t*-test. ^*^P < 0.05 ^**^P < 0.01. (**E**–**F**) Migration (E) and invasion (F) transwell assays were performed in A172 and LN229 cells where H19 expression was knocked-down by siRNAs as in (A–B). The graph shows the number of migrating and invading cells as compared to cells transfected with a non-targeting, negative control siRNA (NC1). Results are presented as mean ± S.D. with significant differences from controls (^*^) shown (*p <* 0.05). Two-tailed unpaired *t*-tests were used to determine significance between groups. The experiments were performed three times (biological replicates).

In order to understand if H19 high expression levels contribute to the oncogenic properties of GBM cells, we depleted H19 by siRNAs (Figure 2A and 2B and Supplementary Figure 1A) and measured cell viability and *in vitro* ability to migrate and invade through an artificial ECM (transwell assays).We assayed three different siRNAs targeting H19, and demonstrated that all of them efficiently knocked down H19 in both A172 and LN229 cells (Supplementary Figure 1B). Due to its high efficiency, we then chose siRNA3 (from now on named “siRNAH19”) for the following experiments. As shown in Figure 2C and Supplementary Figure 1C, H19 knock-down strongly reduced the viability of A172 cells, while it had a more slight, though significant, effect on LN229 cells (Figure 2D). In both cases, the decrease in viability was evident at late time points. Conversely, H19 depletion reduced both migration and invasiveness more in LN229 (Figure 2E and 2F) than in A172 cells, where we observed only a trend (Figure 2E and 2F, and Supplementary Figure 1D). However, even with the differences we detected in our two cell lines, these results indicate that H19 plays a role in the growth and motility of GBM cells. As H19 is also the precursor of miR-675, we also measured miR-675-5p expression in LN229 and A172 cells, before and after H19 silencing. However, in both cell lines we found undetectable levels of miR-675-5p expression (data not shown). This strongly suggests that our functional results in conditions of H19 depletion are not due to miR-675 inhibition, but rather to the inhibition of specific functions played by H19 as a lncRNA.

### H19 localizes at both the cytoplasmic and nuclear region of glioblastoma cells

**Figure 3:**
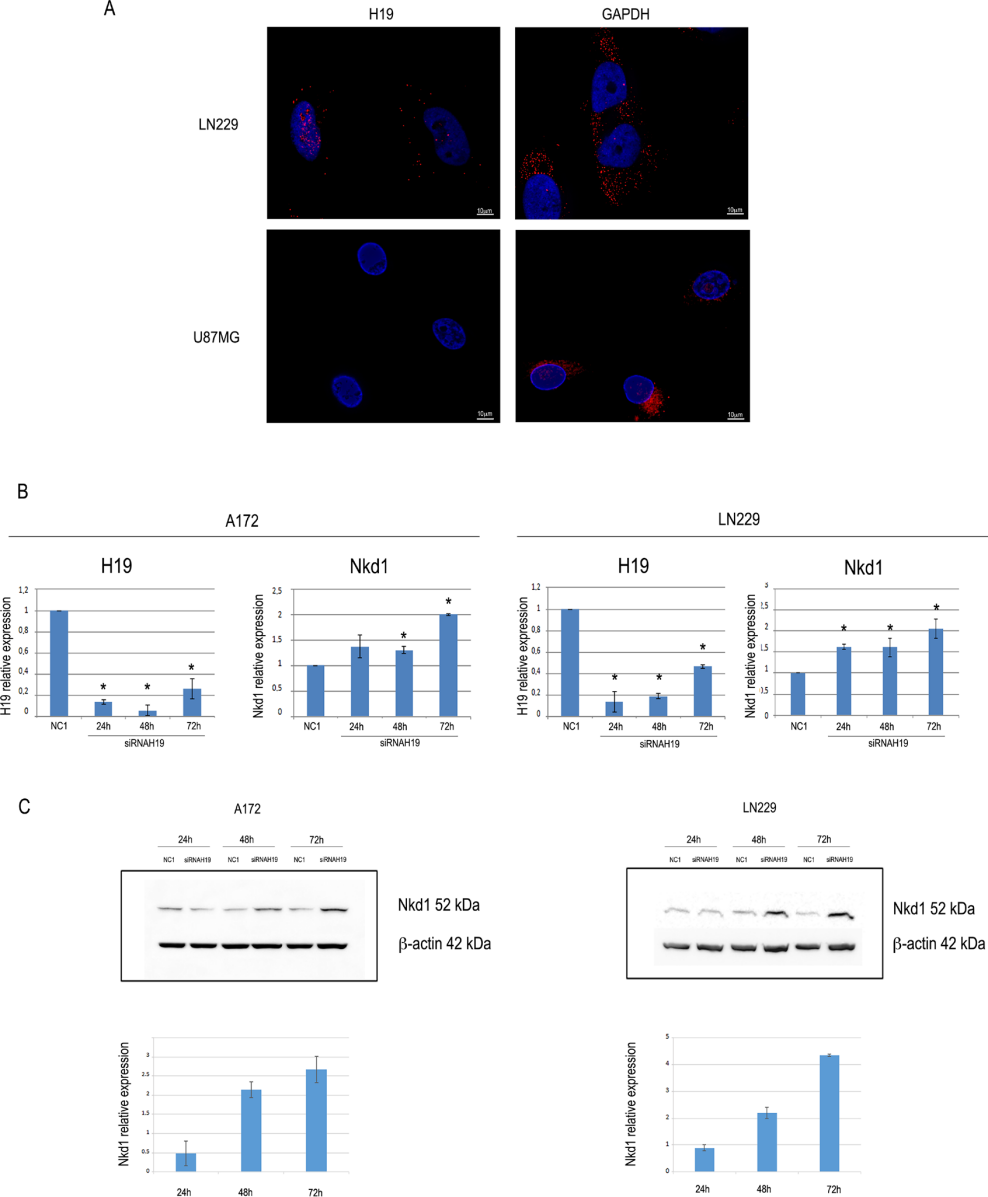
H19 localizes at both nuclear and cytoplasmic regions in LN229 glioblastoma cells, and its knock-down increases the expression of Nkd1 (**A**) Images showing H19 (left) and GAPDH (right) RNA molecules in LN229 and U87MG glioblastoma cells. Nuclei were counterstained by DAPI. Scale bar = 10 μm. (**B**) RTqPCR analysis of NKD1 expression in A172 (left) or LN229 (right) cells transfected with anti-H19 siRNAs. The graph shows the results of RT-qPCR analysis of H19 relative expression in cells transfected with either a non-targeting, negative control siRNA (NC1) or a siRNA specifically targeting H19. At all the time points, H19 and NKD1 expression in siRNA H19-transfected cells is shown as compared to that of NC1-transfected cells, set as = 1. Results are shown as the mean ± S.D. and represent the average of three experiments performed independently. Data were analyzed by a two-tailed unpaired Student’s *t*-test. ^*^P < 0.05. (**C**) The same cells as in (B) were assayed for Nkd1 protein expression by Western blot. Representative Western blots are shown in the upper panels for both A172 (left) and LN229 (right) cell lines, and quantifications are shown in the lower panels, where Nkd1 levels are expressed as the ratio between the silenced samples and the negative control (NC1) at each time point. Beta-actin expression is shown as the loading control.

LncRNAs can play their roles of gene expression regulators in several ways, implying distinct subcellular localizations. As for H19, both cytoplasmic and nuclear roles have been reported [24, 25]; thus, we sought to determine its location in our cells. By employing Stellaris RNA FISH technology [26] to reveal H19 molecules in LN229 cells, we detected their presence both in the cytoplasm and in the nucleus of control cells (Figure 3A). As a further confirmation, we also searched for H19 in U87-MG cells, which did not show any signal, in agreement with our RT-qPCR data (Figure 3A).

### H19 knock-down increases the expression of Nkd1, an inhibitor of the canonical Wnt pathway

Among the roles previously demonstrated for H19 as a modulator of cancer cells, one mechanism of action in the nuclear compartment involves the epigenetic modulation of the expression of Nkd1, an inhibitor of Wnt pathway. In bladder cancer, H19 was shown to direct EZH2 to the promoter region of Nkd1, thus inducing the trimethylation of H3K27 and the consequent repression of transcriptional activity [4]. As the Wnt pathway is strongly involved in glioblastoma [27], we hypothesized that H19 might play the same role in glioblastoma cells. We silenced H19 by transfecting siRNAs into both A172 and LN229 cells (Figure 3B), and we measured Nkd1 mRNA and protein expression. As shown in Figure 3, upon knock-down of H19 both cell lines showed an increase in Nkd1 expression, at the mRNA (Figure 3B) as well as at the protein (Figure 3C) level, suggesting that this lncRNA might affect Nkd1 expression in glioblastoma cells.

### H19 binds to EZH2 in glioblastoma cells and modulates global H3K27 trimethylation

**Figure 4:**
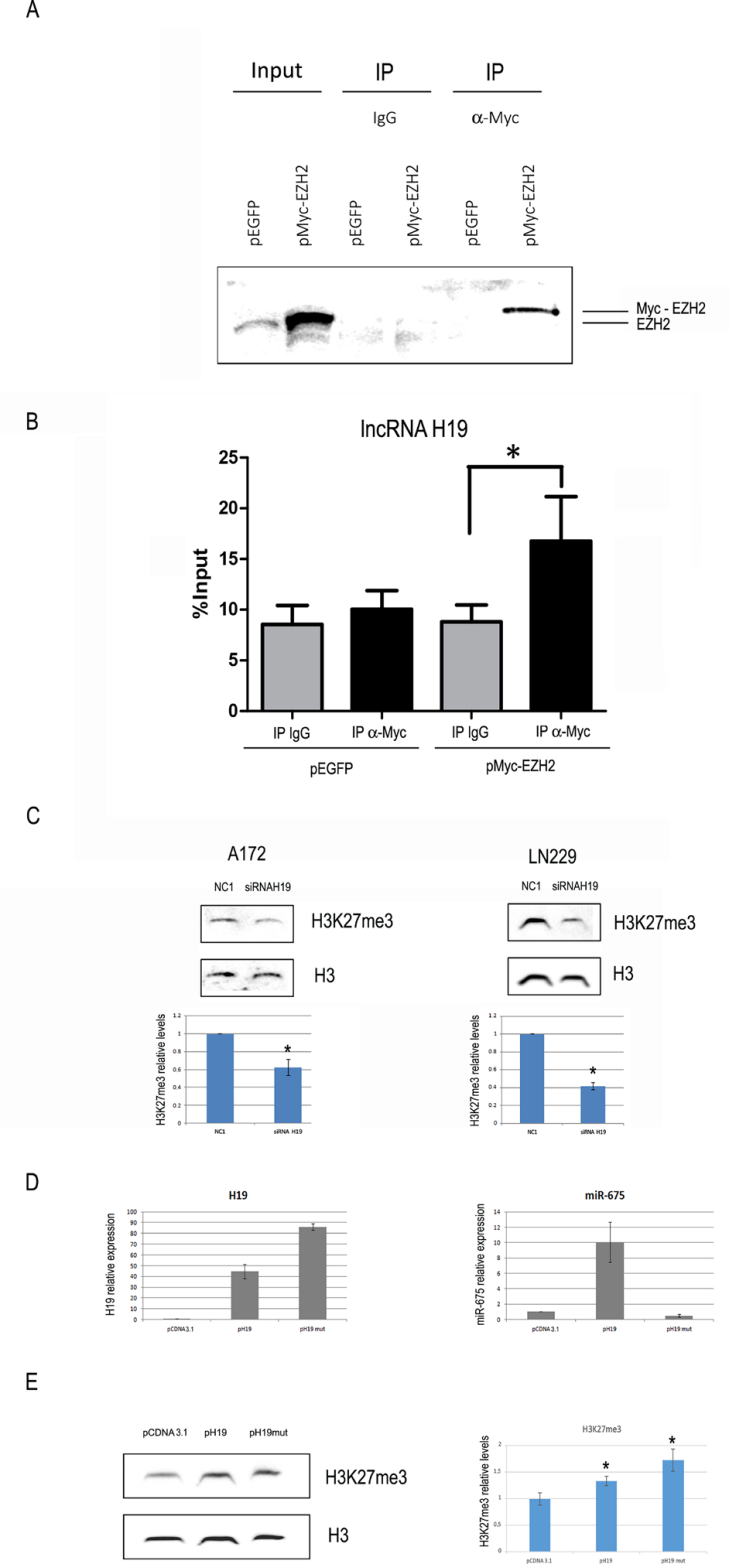
H19 is coimmunoprecipitated with EZH2 and its modulation affects global H3K27 trimethylation in glioblastoma cells (**A**) Cells were transfected with either the negative control vector pEGFP or pMyc-EZH2, a vector encoding a Myc-tagged form of EZH2. Western blot analysis was performed with an antibody against EZH2. The first two lanes (input) show the results of EZH2 WB on total extracts, revealing both endogenous and ectopic EZH2. The remaining lanes, labelled as “IP”, represent proteins immunoprecipitated with either negative control IgGs (third and fourth lanes) or an anti-Myc antibody (fifth and sixth lanes). (**B**) H19 measurement by RT-qPCR on RNA extracted from the IP samples as in (A). Values derived from three independent experiments are reported as means ± s.e.m. and expressed as the percentage of the Input (% Input). Statistically significant differences are reported (^*^P < 0.05). (**C**) Representative western blot analysis (top) and quantification (bottom) of total H3 and H3K27me3 levels in A172 (left) or LN229 (right) cells at 48 h after transfection with either a negative control oligonucleotide (NC1) or an anti-H19 siRNA. Total H3 levels are used as loading controls. (**D**) H19 (left panel) or miR-675-5p (right panel) expression in A172 cells transfected with either the empty vector pCDNA3, or the H19-expressing vector pH19, or the vector pH19mut, encoding for a full length H19 mutated in the miR-675 region. In both panels, data are expressed as compared to pCDNA3-transfected cells, where H19 and miR-675-5p levels are set as = 1. (**E**) Representative western blot analysis (left) and quantification (right) of H3K27me3 and total H3 (loading control) in the same A172 cells as in D. Results in (C–E) are shown as the mean ± S.D. and represent the average of three experiments performed independently. Data were analyzed by a two-tailed unpaired Student’s *t*-test. ^*^P < 0.05.

In order to test if H19 can bind to EZH2 in glioblastoma cells, we performed RNA immunoprecipitation (RIP) experiments in LN229 cells transfected with a vector encoding a myc-tagged EZH2, (or EGFP as control). This analysis showed a statistically significant enrichment of H19 compared to IgGs in the immune precipitated sample of EZH2 expressing only (Figure 4A–4B). The H19-EZH2 interaction was also supported by a computational approach by using the *cat*RAPID algorithm [28], which predicted a good level of protein-RNA interaction propensity (45%) (Supplementary Figure 2).

EZH2 is the catalytic subunit of the PRC2 complex, which works by inducing the trimethylation of H3K27 [29]. We thus analyzed the effects of H19 modulation on H3K27 trimethylation. We silenced H19 by siRNAs and measured H3K27 trimethylation levels by Western blot, showing that it was significantly inhibited in both LN229 and A172 cells (Figure 4C), in agreement with our model where H19 is positively involved in H3K27 trimethylation. In support of this, H3K27me3 levels were increased in A172 cells stably transfected with a vector encoding H19 (Figure 4D–4E). As a further confirmation that H19 plays its role *per se*, and not simply as the precursor of miR-675, we transfected cells also with p-H19-mut, a vector encoding a mutated form of H19, where miR-675 sequence has been mutated and miR-675 is not produced at all (Figure 4D). Even this mutated form of H19, lacking miR-675, was able to increase H3K27 trimethylation (Figure 4E), indicating that miR-675 is not involved in this effect.

### The lncRNA H19 modulates EZH2 binding to the promoter regions of established EZH2 targets

**Figure 5:**
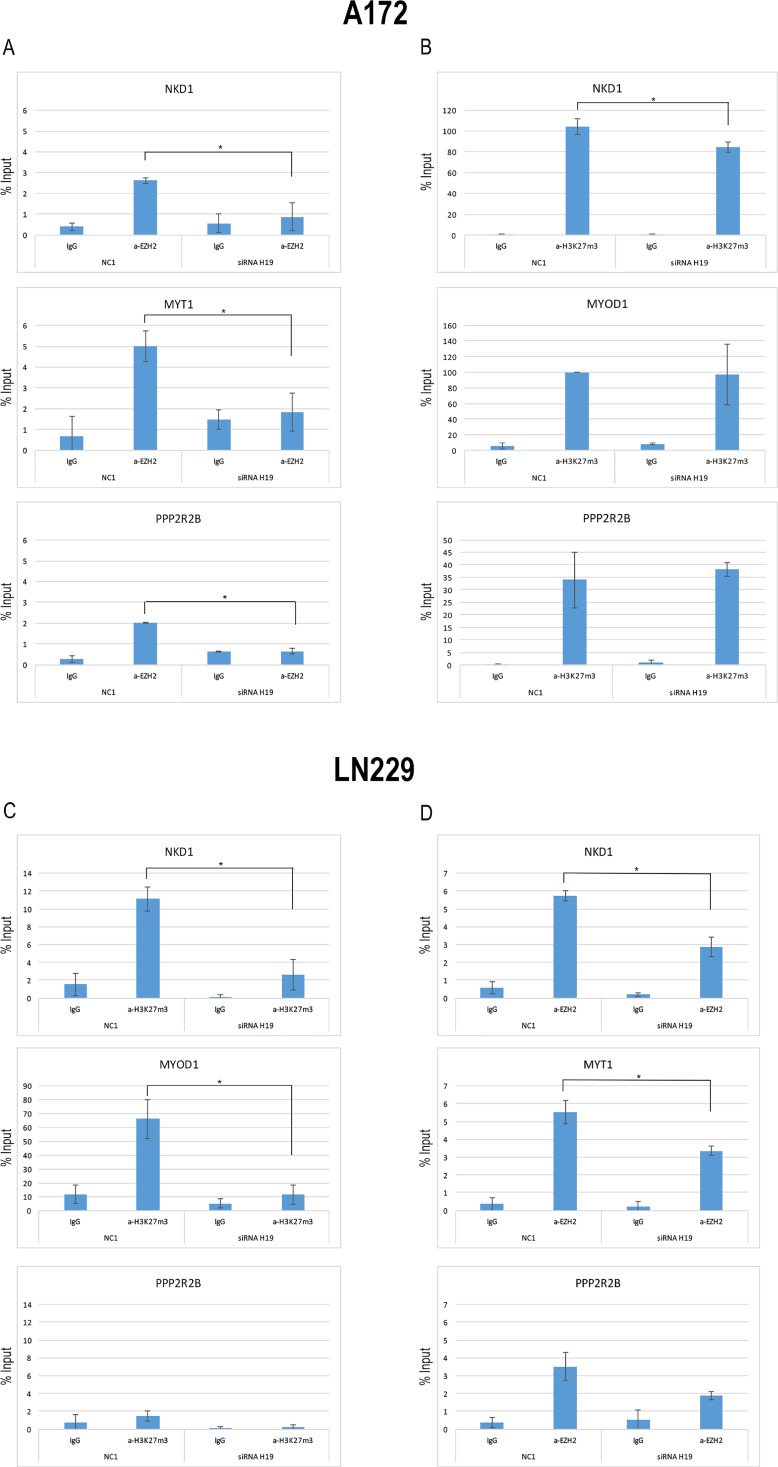
EZH2 is recruited to its target genes by means of H19 (**A**) qPCR analysis of ChIP assays with an anti-EZH2 antibody (a-EZH2) and, as controls, normal rabbit IgG (IgG) on chromatin from A172 cells silenced for H19 (siRNA H19) or with a non targeting siRNA (NC1), as a negative control. Data show the recruitment of EZH2 on the promoters of NKD1 and PPP2R2B, as well as to the regulatory region of MYT1, used as a positive control for EZH2 binding, and its displacement upon H19 knock down. (**B**) qPCR analysis of ChIP assays with anti-H3K27me3 antibody (a-H3K27me3) and, as controls, normal rabbit IgG (IgG) on chromatin from A172 cells silenced for H19 (siRNA H19) or with a non targeting siRNA (NC1), as a negative control. Data show the enrichment of H3K27 trimethylation on the promoter region of NKD1 and its decrease after H19 knockdown. The promoter region of PPP2RB was assayed too, but no H3K27me3 enrichment was found in any condition. The enrichment of H3K27me3 on the regulatory region of MYOD1 is shown as a positive control of the ChIP. (**C**) qPCR analysis of ChIP assays with anti-H3K27me3 antibody (a-H3K27me3) and, as controls, normal rabbit IgG (IgG) on chromatin from LN229 cells silenced for H19 (siRNA H19) or with a non targeting siRNA (NC1), as a negative control. The promoter regions of NKD1 and PPP2R2B were assayed, and the enrichment of H3K27me3 on the regulatory region of MYOD1 is shown as a positive control of the ChIP. (**D**) qPCR analysis of ChIP assays with an anti-EZH2 antibody (a-EZH2) and, as controls, normal rabbit IgG (IgG) on chromatin from LN229 cells silenced for H19 (siRNA H19) or with a non targeting siRNA (NC1), as a negative control. As in (A), the promoter regions of NKD1, PPP2R2B, and MYT1 were assayed. In all ChIP results, values derived from three independent experiments are reported as means ± S.D. and expressed as the percentage of the Input chromatin (% Input). Statistically significant differences are reported (^*^P < 0.05).

We proceeded in our characterization of the role of H19 in glioblastoma cells by investigating if, by modulating H19, we would be able to affect EZH2 binding to promoters of genes known to be epigenetically regulated by PRC2-mediated trimethylation of H3K27 [30]. As shown in Figure 5A, ChIP assays performed in A172 cells knocked-down for H19 revealed that H19 depletion corresponded to a decrease of EZH2 binding to the NKD1 and PPP2R2B promoters, as well as to the regulatory region of MYT1 (which was used as a positive control for EZH2 binding). Moreover, we also showed that H3K27 trimethylation was reduced in the NKD1 promoter upon H19 depletion, while the promoter region of PPP2R2B was not affected in its H3K27 trimethylation levels (Figure 5B).

These results were further confirmed when we repeated the same assays in LN229 cells. Indeed, H3K27me3 was strongly reduced in the NKD1 promoter (Figure 5C). Differently from A172 cells, LN229 did not show a clear enrichment of H3K27me3 marks in the PPP2R2B promoter, and H19 knock-down did not change H3K27me3 levels in that region (Figure 5C). Accordingly, EZH2 binding to NKD1 and MYT1 promoters was reduced by H19 silencing, while it was only slightly affected in the PPP2R2B promoter (Figure 5D).

These results suggest that H19, by binding to EZH2, participates to EZH2 positioning on NKD1 and possibly other promoters, thus maintaining the trimethylated state of H3K27 and the repressed state of the related genes.

## DISCUSSION

The role of H19 as an oncogenic lncRNA is now clearly recognized, and several different modes through which it can promote tumorigenicity in different types of tumors have been demonstrated. The working mechanisms of H19 in cancer can be either epigenetic or post-transcriptional, the former involving the interaction with one or more epigenetic protein modulators, while in the latter H19 sponges specific miRNAs, with a consequent specific target de-repression [3]. In glioblastoma, H19 was first shown to act through its processing into miR-675, in turn repressing Cadherin 13 [8]. An important function was shown for H19 in hypoxia-mediated angiogenesis, again via the production of miR-675, which induces HIF1α nuclear translocation and the consequent angiogenic cascade [12]. Moreover, under hypoxia, HIFα induces H19, which works as a molecular sponge for miR-181d, relieving inhibition of β-catenin expression, and promoting oncogenic effects [31].

In this paper, we take a further step in the understanding of the oncogenic role of H19 in glioblastoma. We show that H19, endogenously expressed in glioblastoma cells, positively influences their growth and ability to migrate and invade. Given that miR-675 is almost undetectable in both A172 and LN229 cell lines, it is unlikely that these functional effects are due to miR-675 expression. We hypothesize that the difference between our results and those described by other Authors [8, 19, 32] may be explained by the different cell lines used, as well as the respective miR-675 expression. Indeed, in the U87 cell line (widely employed in previous papers), we only found very low levels of H19 expression, possibly because in those cells H19 is mostly processed as miR-675 precursor. In agreement with our hypothesis, we also provide evidence of a nuclear role for H19 in LN229 cells, where it is highly expressed: for the first time, we were able to show H19 presence in the nuclear compartment - a finding that corroborates our hypothesis about its role as an epigenetic modulator. We demonstrated that endogenous H19 binds to EZH2, and that its depletion is sufficient to induce a general decrease of H3K27 trimethylation that, again, appears to be independent from miR-675 expression. More specifically, we demonstrated that the depletion of H19 in glioblastoma cells is translated into a specific reduction of EZH2 binding and H3K27 trimethylation at the NKD1 promoter, with a consequent increase of Nkd1 levels. This protein, acting as a cell autonomous inhibitor of the Wnt pathway [33, 34], is involved in the modulation of cancer cell proliferation and migration/invasiveness in several types of solid tumors [30, 35, 36], where its expression is generally reduced compared to healthy tissues. Of note, the promoter region of NKD1 and those of other genes encoding for Wnt inhibitors are frequently hypermethylated in glioblastoma [37], where their expression is consequently very low. Even more directly related to our findings is the observation by Lin B. and colleagues [38] who, by studying H3K4me3 and H3K27me3 profiles in 8 glioblastoma stem cell lines, found that NKD1 promoter contains H3K27me3 marks in 5/8 GBM stem cell lines. This supports and strengthens the results we obtained in established cell lines. We found a further confirmation that H3K27 trimethylation is a common feature contributing to the repressed state of NKD1 not only in cell lines but also in glioblastoma tissues, by data mining in The Canadian Epigenetics, Epigenomics, Environment and Health Research Consortium (CEEHRC). Therein, where H3K27me3 ChIP-seq data from 5 glioma patients’ tissues are deposited, we found that the regulatory region of NKD1 indeed contains peaks of H3K27 trimethylation. The peaks overlap with EZH2 binding sites, and one of them encompasses the region we have used for our ChIP studies on NKD1 regulatory region in the present work (Supplementary Figure 4). All this data, together with the recognized role of the Wnt pathway in glioblastoma [39], suggests a possible importance of NKD1 repression in glioblastoma oncogenesis. In our recent work, where we compared gene expression in glioblastoma tissues to peritumoral regions of the same patients, we observed that, while H19 was clearly overexpressed in tumors, the opposite was true for NKD1 (Log_2_FC peri vs tumor = 1.22, *p* = 0.0086) [21]. We now show for the first time that H19 can regulate NKD1 expression in glioblastoma cells, and we suggest that NKD1 inhibition may be one of the ways through which H19 participates to glioblastoma growth.

Together with NKD1 modulation, we also report for the first time that H19 depletion in A172 glioblastoma cells results in a robust reduction of EZH2 binding to the promoter of MYT1, encoding for Myelin Transcription factor 1. Interestingly, MYT1 is a transcription factor involved in neuronal differentiation, that acts as a repressor of the neural progenitor transcriptional program [40], which is largely shared with that working in glioblastoma initiating cells. However, MYT1 expression remained undetectable in both A172 and LN229 cells even after H19 depletion (data not shown). As MYT1 transcription was shown to be induced by Ascl1, a transcription factor whose expression varies significantly between molecular subtypes of glioblastoma [41], we might speculate that this or other positive signals needed for MYT1 induction may be missing in our cell models, where thus H19 depletion and the consequent reduction of EZH2 binding to MYT1 promoter would not be sufficient to switch its transcription on.

Our results point to a role for H19 as a component of an epigenetic regulatory complex in glioblastoma cells. H19 would cooperate with EZH2 in its repressive function on specific promoters, either driving or maintaining the PRC2 complex on targets. The interaction of H19 with the EZH2-centered regulation has been previously demonstrated in bladder carcinoma, where a mechanism analogous to the one hypothesized in this paper was proposed [4], and in ERα-positive breast cancer, contributing to chemoresistance [42]. Much more generally, H19 was shown to induce an increase of EZH2 levels in nasopharyngeal carcinoma cells, by working as a “sponge” for miR-630, thus sequestering it from its target, EZH2 mRNA [43]. We propose that H19 might contribute in more than one way to the EZH2-mediated repression of key anti-tumor genes. Depending on the cell- and tumor specific context, either the nuclear function acting on specific promoters or the cytoplasmic, “sponge” role may prevail, with the two mechanisms very likely coexisting. Indeed, in the glioblastoma cell lines we have tested, we did not find evidence of a possible general effect of H19 depletion on EZH2 protein levels (Supplementary Figure 3A–3B). Moreover, in both cell lines the levels of miR-630 were close to undetectable (data not shown), thus not supporting the “sponge” role for H19 in these cells. In conclusion, in this work we describe H19 as part of an epigenetic modulation mechanism centered on EZH2, leading to the repression of NKD1, a gene involved in the negative regulation of the Wnt pathway. The here provided evidence unveils a new perspective on the role of this oncogenic lncRNA in glioblastoma.

## MATERIALS AND METHODS

### Cell lines, treatments and transfection

A172, LN229, U87MG, LN18 and T98G cell lines (ATCC, Rockville, MD, USA) were cultured in Dulbecco’s modified Eagle’s medium supplemented with 10% fetal bovine serum (Aurogene) in a humidified atmosphere containing 5% CO_2_ at 37° C. Transfections were performed by Lipofectamine 3000 reagent (Invitrogen) using plasmid DNA or *DsiRNA* H19 (HSC.RNAI.NR_002196.12, which consists of 3 predesigned DsiRNAs for the same target, H19, a negative control, NC1, and positive controls for transfection, TYE, all from Integrated DNA Technologies, IDT) in Opti-MEM I (Invitrogen), as recommended by the manufacturer.

Plasmids used: pcDNA3.1 empty vector (Clontech); pEGFP-C1 empty vector (Clontech); pCMV-Script H19 (BamH1/EcoRI) kindly provided by Dr. Imad Matouk, the Hebrew University of Jerusalem, Israel; pCMV-Script H19 mut (BamH1/EcoRI) was generated by mutating the miR-675 sequence within H19 (3 nucleotides in the seed sequence of the miR and 3 nucleotides outside the seed). The primers used for mutagenesis were the following:

For: 5′ tgtacaggcgaggtccgacagtggacttgg 3′

Rev: 5′ tgtcggacctcgcctgtacagaccctgggc 3′

p-Myc-EZH2 vector was kindly provided by Dr.ssa Daniela Palacios, Fondazione Santa Lucia IRCCS, Roma.

The generation of stably transfected A172 cell lines was obtained by growing cells in medium containing the selective agent, G418, 1 mg/ml for three weeks.

### RNA isolation, reverse transcription and Quantitative Real Time Polymerase Chain Reaction (RT-qPCR)

Total cellular RNA was harvested with TRIzol LS Reagent^®^ (Life Technologies), treated with DNase (Biolabs) for 40 min at 37° C, and reverse transcribed with M-MLV Reverse Transcriptase (Invitrogen), according to the manufacturer’s protocols. RT-qPCR was performed on the Applied Biosystems StepOne plus PCR System with PowerUp™ SYBR Green Master Mix (Life Technologies). The relative amount of each substrate was calculated by the 2^–ΔΔCT^ method.

NKD1 and mir-675 expressions were measured by qRT-PCRs conducted on a StepOnePlus Real-Time PCR System (Applied Biosystems-Life Technologies) using TaqMan Universal PCR Master Mix and the specific TaqMan^®^ Assays (probe and primer sets) (Applied Biosystems-Life Technologies). The small endogenous nuclear RNA U6 (RNU6B) and TBP were used as controls for normalization. TBP, ID Hs99999910_m1; NKD1, Hs00263894_m1; RNU6B, ID 001093; hsa-miR-675-5p, 121124_mat.

The primers used in RT–qPCR analysis with SYBR Green Master Mix are listed in Table 2.

**Table 2:** List of primers used in RT-qPCR analysis

Target gene	Forward primer	Reverse primer
H19	5′CTGGGCAACGGAGGTGTA3′	5′CTGGGAGGGTGTCTGCTTC3′
beta-ACTIN	5′ACCGAGCGCGGCTACAG3′	5′CTTAATGTCACGCACGATTTC3′

### RNA immunoprecipitation (RIP)

RIP was performed starting from 1 mg of cleared lysate. Cell lysates were precleared with 20 µl of Dynabeads Magnetic (Invitrogen) and then were incubated with 3 µg of antibody in PBS Tween 0.02% for 2 h at 4° C in rotation. Furthermore, 30 µl of Dynabeads Magnetic were added to the protein extract and incubated 1 h at 4° C in rotation. Protein-RNA complexes bound to beads were washed three times in NP-40 buffer (150 mM NaCl, 50 mMTris-HCl pH 8, 0.5% NP40, 10% glycerol, protease inhibitor cocktail and RNase inhibitor). The immunoprecipitates were divided in two parts for protein and RNA recovery. Proteins were eluted from beads by Laemmli sample buffer at 75° C 10 minutes. Co-precipitated RNA was treated 20 minutes at 37° C with proteinase K and recovered by phenol-chloroform extraction and ethanol precipitation and subjected to RT-PCR analysis. Primary antibodies for IP were mouse monoclonal anti-Myc (sc-40 Santa Cruz) or mouse IgG (Invitrogen).

### Western blot analysis

For Western blot analysis, total protein extract was isolated from A172 and LN229 cells by lysis in NP-40 Buffer containing protease and phosphatase inhibitors (complete EDTA-free; Roche Applied Science), and protein concentrations were determined by Bradford method. Equivalent amounts of protein extract were separated by electrophoresis on 10% or 12% SDS-PAGE gels and blotted onto nitrocellulose. The membranes were blocked with 5% non-fat dry milk and 0.1% Tween-20 in Phosphate-buffered saline and then incubated with antibodies followed by appropriate horseradish peroxidase-conjugated secondary antibodies (Promega). Rabbit monoclonal anti-NKD1 (OriGene) was used diluted 1:4000, mouse monoclonal anti-EZH2 (Cell Signaling) was diluted 1:1000, mouse monoclonal anti-Myc (Santa Cruz) was diluted 1:1000, mouse monoclonal H3 (Abcam) was diluted 1:1000, rabbit polyclonal H3K27me3 (Millipore) was diluted 1:10000. Rabbit polyclonal anti-β-actin (Sigma) diluted 1:5000 was used to reveal β-actin as a loading control.

### Histone protein extraction

Cells were harvested and washed twice with ice-cold PBS. Cells were resuspended in Triton Extraction Buffer (TEB: PBS containing 0.5% Triton X 100, protease inhibitors) at a cell density of 10^7^ cells per ml and incubated on ice for 10 minutes with gentle shaking. After centrifuging at 3000 rpm for 10 minutes at 4° C, the supernatant was removed and discarded. The pellet was resuspended in 25 µl 0.5N HCl and incubated on ice for 30 minutes. 4 volumes of pre-chilled acetone was added to the sample, and histones were let precipitate at –20° C overnight. After centrifuging at 13000 rpm for 20 minutes at 4° C, the supernatant was removed and the pellet air-dried and resuspended in distilled water. Protein content was determined using the Bradford assay.

### Chromatin immunoprecipitation (ChIP) analysis

After cross-linking with 1% formaldehyde, cultured cells 48 h post transfection were scraped off in ice-cold phosphate-buffered saline plus protease and phosphatase inhibitors, centrifuged at 2000 rpm for 5 minutes at 4° C and pellets resuspended in L1 Buffer (50 mM TrisHCl pH 8.0, 2 mM EDTA pH 8.0, 0.1% NP-40, 10%, glycerol plus protease and phosphatase inhibitors) for 15 minutes at 4° C in rotation. The lysates were homogenized by 15 douncer strokes and then centrifuged at 5000 rpm for 15 minutes at 4° C, to separate the cytoplasmic from the nuclear fraction. The pellets, containing nuclei, were resuspended in L2 buffer (50 mM TrisHCl pH 8.0, 5 mM EDTA pH 8.0, 1% SDS plus protease and phosphatase inhibitors) and incubated for 20 minutes at 4° C in rotation. The chromatin was sonicated on ice with 10 pulses for 10 seconds at 70% settings to obtain chromatin fragments of an average length of 300 to 500 base-pairs. Successively, chromatin was centrifuged at 10.000 rpm for 10 minutes, and supernatants were collected. For each sample, two 130 μg aliquots (one for specific antibody and one for the species-specific corresponding IgG) were diluted 1:10 in Dilution Buffer (50 mM TrisHCl pH 8.0, 200 mM NaCl, 5 mM EDTA, 0.5% NP-40) plus protease inhibitors, and precleared with 40 μl of *Protein A Sepharose* (Sigma Aldrich) (previously blocked with sonicated salmon sperm DNA 200 μg/ml in 3% Bovine Serum Albumin) for 3 h at 4° C in rotation. Pre-cleared chromatin was centrifuged at 13000 rpm for 5 minutes and the supernatant was incubated over night at 4° C in rotation with 5 μg of mouse monoclonal α-EZH2 or the negative control mouse IgG (ChIPAb+EZH2, clone AC22, Millipore), to proceed with immunoprecipitation. Immunoprecipitated complexes were collected by incubation with 50 μl of Protein A Sepharose for 3 hours at 4° C in rotation. The samples were centrifuged at 3000 rpm for 5 minutes at 4° C and, before washing, 300 μl of the supernatant of the IgG sample were collected and stored as the Input sample.

Then the beads were washed in the following buffers with protease and phosphatase inhibitors:

- Low salt (20 mM TrisHCl pH 8.0, 150 mM NaCl, 2 mM EDTA, 0.1% SDS, 1% TritonX-100)

- High salt Buffer (20 mM TrisHCl pH 8.0, 500 mM NaCl, 2 mM EDTA, 0.1% SDS, 1% Triton X-100)

- LiCl wash Buffer (10 mM TrisHCl pH 8.0, 0.25M LiCl, 1% NP-40, 1% deoxycholate, 1mM EDTA)

- TE wash Buffer (10 mM TrisHCl pH 8.0, 1 mM EDTA pH 8.0) After that, immune-complexes were eluted twice from *Protein A Sepharose* with 150 μl of Elution buffer (1% SDS and 100 mM NaHCO_3_) for 15 minutes with shaking at RT.

Then the samples were incubated with 10 μg of RNase for 10 minutes at RT and after that, cross-linking was reversed by incubating samples at 67° C over-night with gentle shaking. Each sample was supplemented with 6 μl of 1M Tris-HCl pH 6.5 to neutralize NaHCO_3_, and then 12 μl of proteinase K 20 mg/ml was added and the reaction conducted for 2 hours at 56° C. Finally, DNA was extracted with phenol-chloroform and chloroform and then precipitated in 1 volume of 100% isopropanol. The pellet was washed with cold 70% ethanol and resuspended in 50 μl of H_2_0 and chromatin concentration was determined. Equal amounts of immunoprecipitated DNA and relative controls were used for qPCR analysis. qPCR analysis of immunoprecipitated samples (IP) and negative control (IgG) were normalized to total chromatin input and expressed as (2^( –ΔCt)^) × 100 (% Input).

Primers employed for ChIP are reported in Table 3.

**Table 3:** List of primers used in ChIP analysis of the DNA

Target gene	Forward primer	Reverse primer
MYT1	5′ACAAAGGCAGATACCCAACG3′	5′GCAGTTTCAAAAAGCCATCC3′
PPP2R2B	5′CTGGGCAGCAAGCGACTAG3′	5′TAGACGTGGCCCTTAGCTGAGT3′
MYOD1	5′CCGCCTGAGCAAAGTAAATGA3′	5′GGCAACCGCTGGTTTGG3′
NKD1	5′AGATCCCAGAGACTGGCTGATAAC3′	5′CGCACTCCTCTAGTTTCTTATTTGG3′

### Chromatin immunoprecipitation of H3K27me3

H3K27me3 ChIP analysis was performed by using 5 μg of mouse monoclonal H3K27me3 antibody (07-449; Millipore Corp.) or mouse IgG (Millipore Corp.) with Dynabeads Magnetic (Invitrogen). After washes, samples were eluted with the elution buffer (TE 1×, sodium dodecyl sulfate 0.5%), treated with RNase A and with proteinase K. DNA was extracted with phenol-chloroform. Equal amounts of immunoprecipitated DNA and relative controls were used for qPCR analysis. qPCR analysis of immunoprecipitated samples (IP) and negative control (IgG) were normalized to total chromatin input and expressed as (2^( −ΔCt)^) × 100 (% Input).

### RNA *in situ* hybridization

Cells were grown on 24 × 24 mm coverglasses in a 6-well cell culture plate. Stellaris^®^ RNA FISH Probes (Biosearch^®^ Technologies) were used for the detection of RNA according to the manufacturer’s protocol for adherent cells. The probes were incubated in the dark at 37° C for 4 hours. Coverglasses were mounted by placing a drop of ProLong^®^ Diamond Antifade Mountant with DAPI (Life Technologies). The probes used were: Human H19 with Quasar^®^ 570 Dye or Human GAPDH with Quasar^®^ 570 Dye. The microscope used to capture images was Leica DMI 6000B.

### Migration and invasion assays

A172 and LN229 cells were plated in DMEM culture medium without serum on BD BioCoat Control Cell Culture Insert Systems or on BD BioCoat Matrigel Invasion Chamber (BD Bioscience) at 25 × 10^3^ cells/chamber for migration or invasion assays, respectively. The chemoattractant (DMEM supplemented with 10% FBS) was added to the bottom wells of the plate.

The cells were incubated at 37° C, 5% CO_2_, for 6 h for migration and 24 h for invasion. After incubation, the non-migrated/invaded cells were removed from the upper surface of the membrane by scrubbing with a cotton tipped swab, while the cells migrated/invaded adhering to the bottom surface of the membrane were fixed with 100% MetOH and stained with 0.1% crystal violet, 3% EtOH. The number of migrated or invaded cells was evaluated in three different fields of three different wells for each condition.

### MTS cell viability analysis

A172 and LN229 cells were trypsinized, harvested and seeded onto 96-well flat-bottomed plates at a density of 2,000 cells/well, then incubated at 37° C for 24 h in DMEM supplemented with 10% FBS. Subsequently, the cells were subjected to CellTiter 96^®^ AQueous One Solution Cell Proliferation Assay (Promega), according to the manufacturer’s protocol. The absorbance at 490 nm was evaluated using a BP800 Microplate Absorbance Reader (Headquarters BiohitOyj).

### Statistical analysis

Paired *t*-test and GraphPad Prism version 5.00 (GraphPad software, San Diego, CA, USA; http://www.graphpad.com) were used. A *p*-value (*P*) < 0.05 was considered statistically significant. Data were obtained from independent experiments (*n* = 3) expressed as means ± SD. Factor analysis with varimax rotation was performed in Statistica (Statsoft) v. 10.

## SUPPLEMENTARY MATERIALS FIGURES AND TABLE




