# Supplementary material for: The lncRNA H19 positively affects the tumorigenic properties of glioblastoma cells and contributes to NKD1 repression through the recruitment of EZH2 on its promoter
Source: Oncotarget. 2018 Feb 14;9(21):15512–25. doi: 10.18632/oncotarget.24496 (PMC5884644; doi:10.18632/oncotarget.24496)
Supplement: Supplementary file 1 [file oncotarget-09-15512-s001.pdf]

The lncRNA H19 positively affects the tumorigenic properties of glioblastoma cells and contributes to NKD1 repression through the recruitment of EZH2 on its promoter

SUPPLEMENTARY MATERIALS

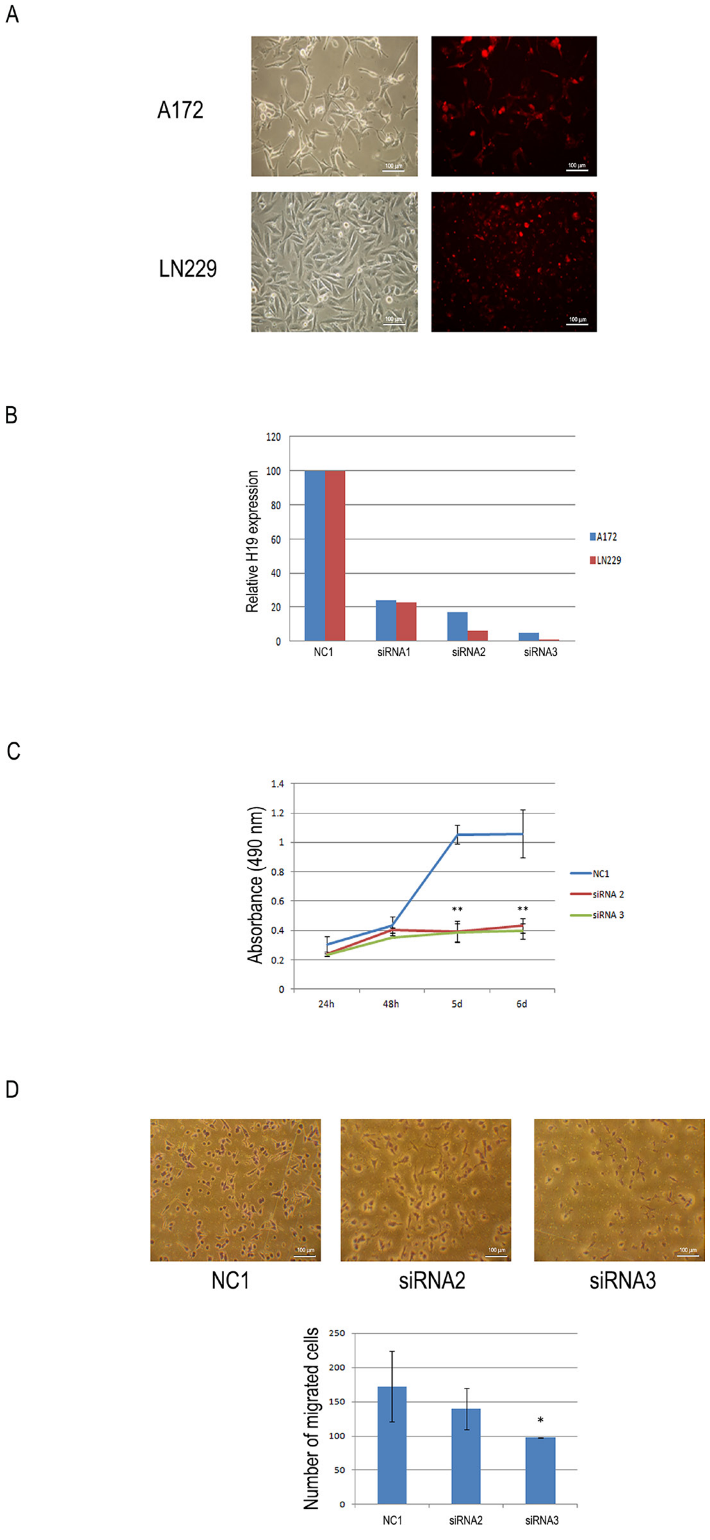

Supplementary Figure 1: (**A**) Representative images of A172 and LN229 cells transfected with a fluorescently labelled control siRNA. Scale bar = 100 µm. (**B**) H19 knock down by three distinct siRNAs in A172 and LN229 cells. The graph shows the results of a RT-qPCR analysis of H19 relative expression in cells transfected with either a non-targeting, negative control siRNA (NC1) or three different siRNAs specifically targeting H19, at 24 hours after transfection.H19 expression in siRNA H19-transfected cells is shown as compared to that of NC1-transfected cells, set as = 100%. The figure shows the results of one representative experiment. (**C**) Viability (MTS) assays performed on A172 cells transfected with either a negative control (NC1) or two different H19 targeting siRNAs, as in (B). Results are shown as the mean ± S.D. and represent the average of two experiments performed independently. Data were analyzed by a two-tailed unpaired Student's *t*-test. $^{**}P < 0.01$. (D) Migration transwell assay performed in A172 cells where H19 expression was knocked-down by siRNAs as in (B). The upper panel shows representative images of migrated cells in each of the three different conditions. The graph shows the number of migrated cells as compared to cells transfected with a non-targeting, negative control siRNA (NC1). Results are presented as mean ± S.D. Data were analyzed by a two-tailed unpaired Student's *t*-test. $^{*}P < 0.05$. The results are from one representative experiment performed in triplicate.

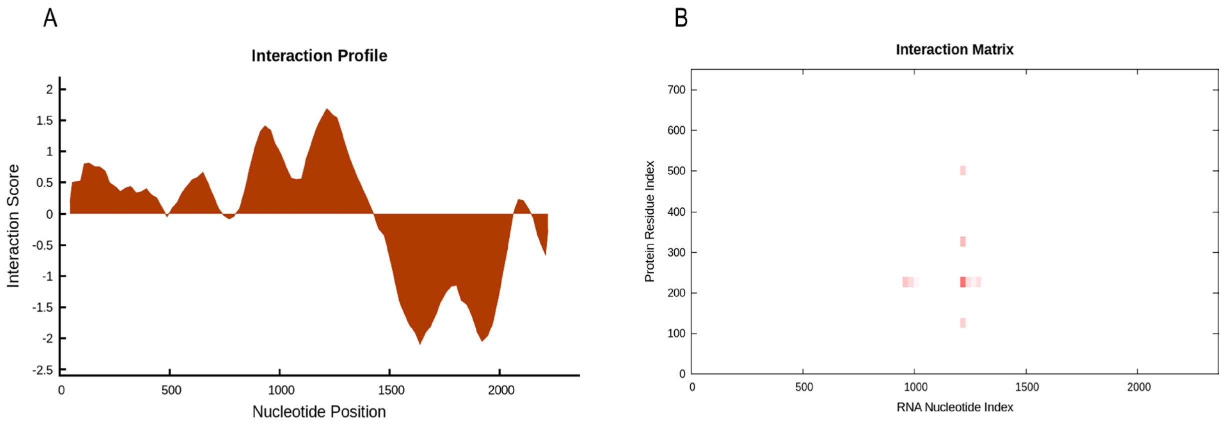

Supplementary Figure 2: catRAPID prediction analysis of the potential RNA-protein interactions between H19 and EZH2. This method is able to predict protein-RNA interaction propensities with great accuracy through the calculation of secondary structure, hydrogen bonding and van der Waals contributions. The EZH2 and H19 sequences employed for the prediction were NCBI reference sequences NP_004447.2 and NR_002196.2, respectively. Panels a and b show the interaction profile and the interaction matrix predicted for EZH2-H19 interaction, respectively.

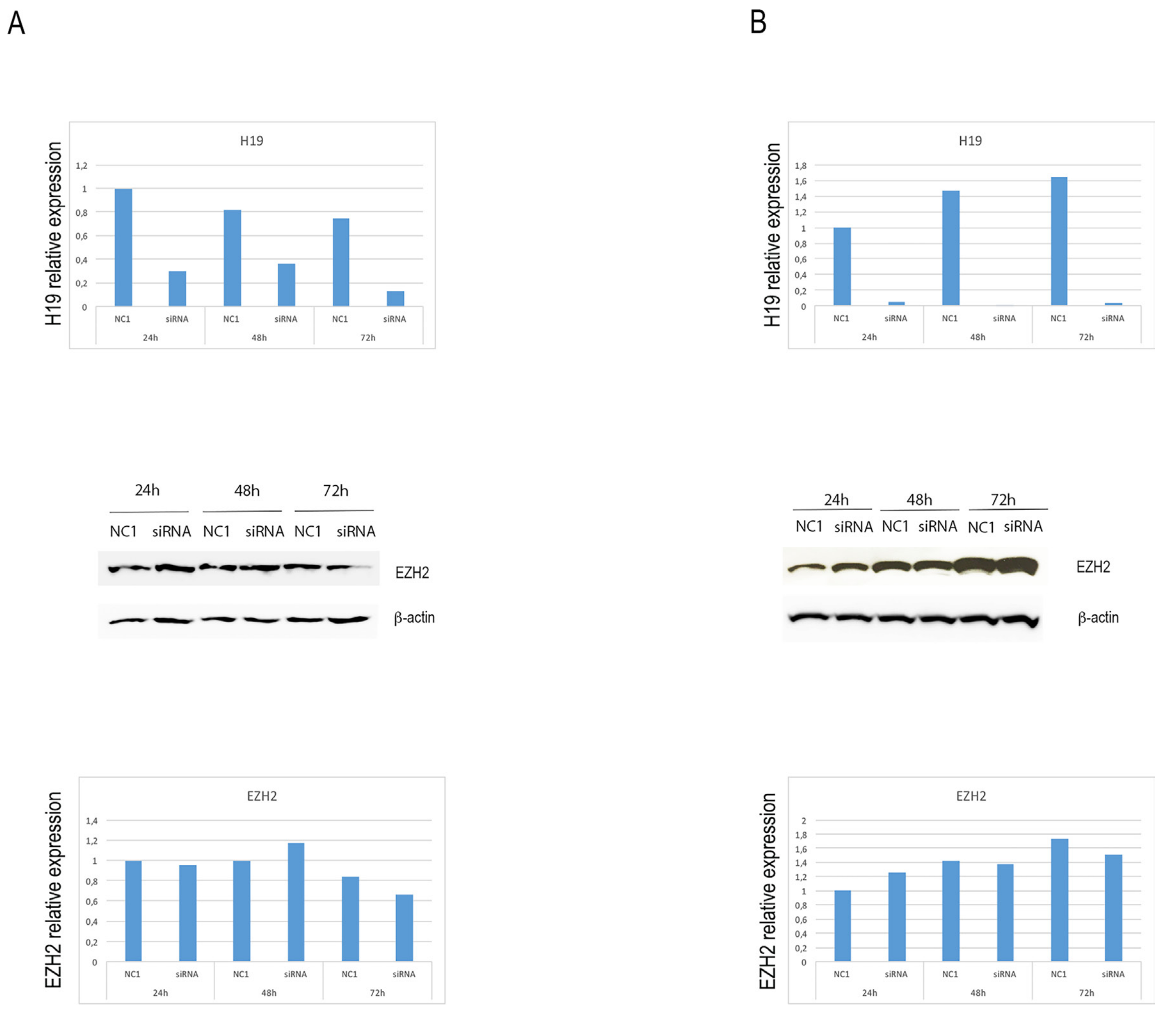

Supplementary Figure 3: Graphs in A and B show the results of an H19 silencing experiment in A172 and LN229 cells, respectively, at 24, 48 and 72 hours after transfection with anti-H19 siRNAs or with the negative control siRNA NC1. The middle panels show a Western blot analysis of EZH2 protein levels in the same A172 (left) or LN229 (right) cells. Beta-actin was used as the loading control. The lower panels show the densitometric quantification of EZH2 normalized by beta-actin expression.

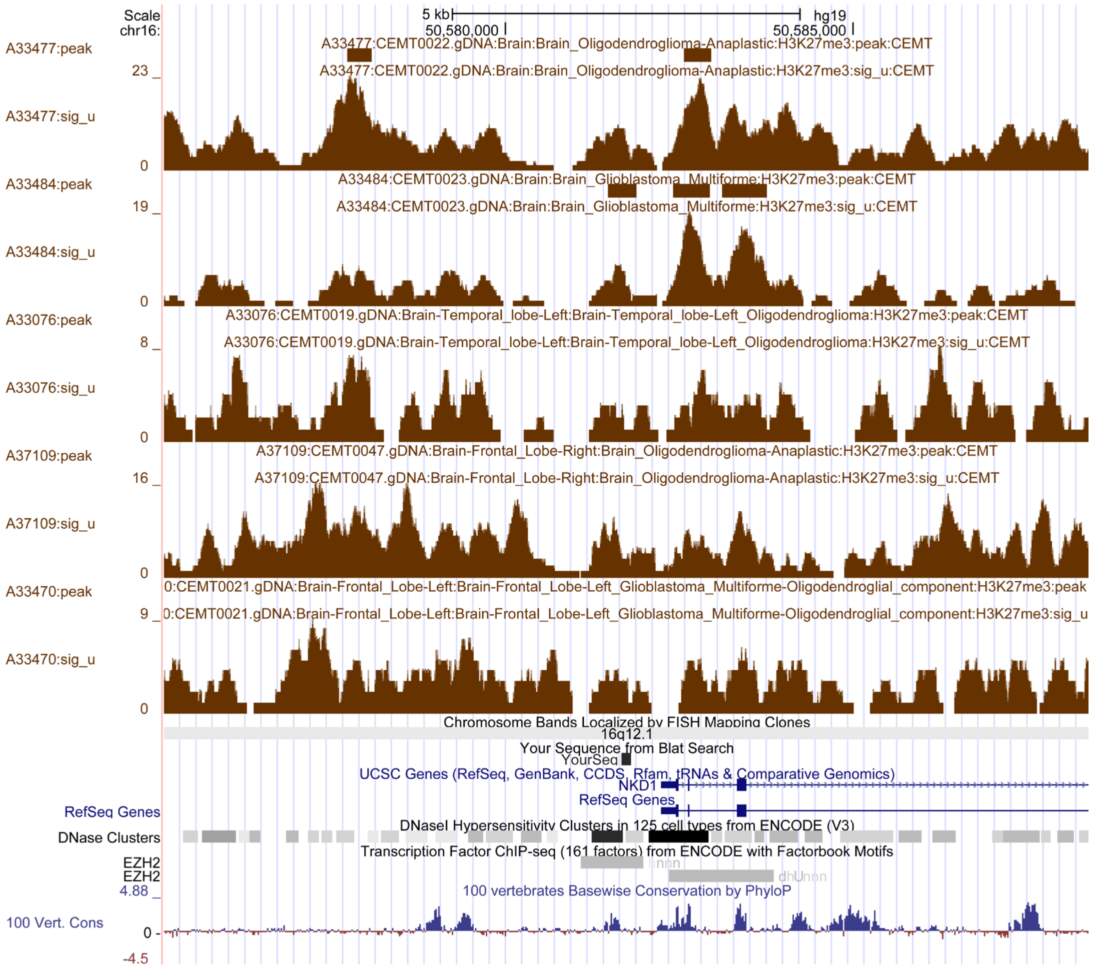

Supplementary Figure 4: A screenshot from UCSC Genome Browser (GRCh37/hg19) Assembly showing genomic coordinates chr16:50,575,138-50,588,377, encompassing the regulatory region and the first part of NKD1 transcript. The graph shows signals and relative peaks of H3K27me3 ChIP in five tissues from glioma patients. Also shown are sites of DNAse hypersensitivity, regions of EZH2 binding from ChIP-seq experiments, and the region of NKD1 promoter (named "Your Seq") amplified in our ChIP experiments. The results shown in this Figure are in part based upon data generated by The Canadian Epigenetics, Epigenomics, Environment and Health Research Consortium (CEEHRC) initiative funded by the Canadian Institutes of Health Research (CIHR), Genome BC, and Genome Quebec. Information about CEEHRC and the participating investigators and institutions can be found at http://www.cihr-irsc.gc.ca/e/43734.html.

Supplementary Table 1: Complete factor analysis results of 12 glioblastoma tissues, described in ref.21. See Supplementary_Table_1
